# Esophageal temperature during atrial fibrillation ablation poorly predicts esophageal injury: An observational study

**DOI:** 10.1016/j.hroo.2021.11.002

**Published:** 2021-11-05

**Authors:** Tarek Ayoub, Abdel Hadi El Hajjar, Gursukhman Deep Singh Sidhu, Arezu Bhatnagar, Yichi Zhang, Mario Mekhael, Charbel Noujaim, Lilas Dagher, Christopher Pottle, Nassir Marrouche

**Affiliations:** Tulane Research and Innovation for Arrhythmia Discoveries – TRIAD Center, Tulane University School of Medicine, New Orleans, Louisiana

**Keywords:** Atrial fibrillation, Catheter ablation, Esophageal injury, Esophageal temperature, Left atrium, Pulmonary vein isolation

## Abstract

**Background:**

Esophageal injury (EI) remains a concern when performing pulmonary vein isolation (PVI) using the high-power short-duration (HPSD) technique.

**Objective:**

We aim to indicate that high esophageal temperature during HPSD PVI does not correlate with positive esophageal endoscopy (EGD) findings.

**Methods:**

A retrospective observational study was performed on 43 patients undergoing PVI using HPSD (50 W for 6–7 seconds per lesion) at Tulane Medical Center from July 2020 to January 2021. Esophageal temperature was monitored throughout the procedure using a temperature probe and patients underwent EGD the following day. Small ulcers, nonbleeding erosions, erythema, and/or esophagitis were considered positive EGD findings.

**Results:**

Mean age was 64.9 years; 46.5% of the patients were female. Eleven patients had positive EGD findings (group 1) and 32 patients had normal EGD (group 2). There was no statistical difference in mean esophageal peak temperature between group 1 and group 2 (43.9°C ± 2.9°C and 42.5°C ± 2.3°C, respectively, *P* = .17). There was no association between positive EGD results and esophageal temperature during PVI. Mean baseline esophageal temperature was similar in both groups (36.1°C, *P* = .78). Average contact force (*P* = .53), ablation time (*P* = .67), age (*P* = .3096), sex (*P* = .4), body mass index (*P* = .14), and other comorbidities did not correlate with positive endoscopy results. We found positive correlation between the distance of the left atrium (LA) to esophagus and positive EGD (*P* = .0001).

**Conclusion:**

EI during HPSD PVI does not correlate to esophageal temperature changes during ablation. However, esophageal injury does correlate to a shorter proximity of the esophagus to the LA.

**Key Findings**

▪ When monitoring was applied, no correlation was seen between esophageal injury during pulmonary vein isolation ablation and esophageal temperature changes.
▪ The lack of an increase in temperature does not guarantee esophageal protection.
▪ A small distance between the left atrium and the esophagus is the main risk factor for esophageal injury after atrial fibrillation ablation.

## Introduction

High-power short-duration (HPSD) application of radiofrequency (RF) current for pulmonary vein isolation (PVI) is a safe and effective technique with increasing utilization^1^, ^2^, ^3^ for the treatment of atrial fibrillation (AF). The advantages of HPSD RF PVI include improved resistive heating, reduced thermal latency, and conductive collateral tissue injury.^4^^,^^5^ Factors such as conductive heating, local inflammation, and postablation reflux mechanisms play significant roles in causing esophageal injury (EI). Atrioesophageal fistula (AEF) is a rare esophageal complication (incidence rate of 0.1%–0.25%) associated with this procedure. However, its mortality rate has been reported to be as high as 80%.^6^ Thus, in order to prevent AEF, an established method has been to avoid conducting cardiac ablations near to the esophagus. Given the anatomical position of the esophagus and its proximity to the posterior atrial wall and the right lower pulmonary vein, ablation around this region is often necessary to achieve a successful outcome.^7^

Deep tissue heating during catheter ablation can be controlled via different approaches. Essentially, the energy delivered can be calculated as the amount of power delivered to the tissue multiplied by time. Compared to the conventional low-power, longer-duration (LPLD) approach, HPSD offers more accurate therapeutic lesions and a decreased depth penetration secondary to thermal latency reduction.^1^, ^2^, ^3^, ^4^, ^5^, ^6^

Currently, adequate evidence linking esophageal thermal injury to HPSD ablation procedures is lacking and should be investigated further. Esophageal temperature monitoring is one way to reduce the risk of thermal injury intraoperatively.^8^ However, this method has mostly been researched using LPLD ablation procedures.^7^^,^^9^^,^^10^ Thus, the means by which HPSD may result in EI remains unclear.

For this reason, our observational study evaluated the correlation between esophageal lesions seen during esophageal endoscopy (esophagogastroduodenoscopy; EGD) post-PVI and compared it to intraoperative esophageal temperatures achieved during HPSD PVI. We also took into consideration other factors that could increase the risk of injury, eg, average force used, power, and total time of procedure, and compared it with any observed esophageal lesions. In addition, a separate analysis measuring the distance between the left atrium (LA) and the esophagus (LA-esophageal) was also performed on a subset of patients.

## Methods

### Study design

This retrospective observational study aimed to associate positive EGD findings after HPSD ablation when esophageal temperatures could be high.

### Patient population

From July 2020 to January 2021, 43 AF patients (paroxysmal or persistent type) who underwent a first-time AF ablation with next-day EGD were included in this study. Demographic information, clinical history, and imaging data were all collected from the Tulane Research Innovation for Arrhythmia Discoveries (TRIAD) database, a registry that includes AF patients undergoing cardiac ablation at Tulane Medical Center, New Orleans, LA. On December 13, 2019, the Tulane University Biomedical Institutional Review Board provided an expedited review and approval determination for the initial submission of this minimal risk study. Patient consent was waived owing to the use of retrospective and de-identified data. The review was provided in accordance with the appropriate research regulations. The research reported in this paper adhered to the Helsinki Declaration guidelines.

### Ablation procedure

Patients undergoing catheter ablation for AF were placed on anticoagulant therapy at least 3 weeks prior to their procedure. Signed informed consent forms were also obtained prior to cardiac ablation.

All patients included in the study underwent an RF ablation procedure in accordance with the latest guidelines.^11^ The procedure was performed using a 3-dimensional electroanatomic mapping system (CARTO; Biosense Webster, Irvine, CA). After achieving transseptal access, patients received 10,000 IU of heparin and had activated clotting time monitored every 15 minutes to maintain it between 300 and 350 seconds.

The ablation consisted of isolating the pulmonary veins first, followed by creating an ablation line at the roofline, which connects both left- and right-sided lesions. Finally, fibrosis-targeted ablation was performed in all patients, mainly on the posterior atrial wall, guided by magnetic resonance imaging (MRI) images or mapping images. RF energy was delivered using an irrigated RF ablation catheter (ThermoCool SmartTouch; Biosense Webster) at a distance of 10 mm from the pulmonary vein (PV) ostia using HPSD ablation technique (50 W for 5–7 seconds per lesion, temperature 50°C). PVI was assessed continuously in all pulmonary veins using the CARTO® PENTARAY™ NAV eco Catheter (Biosense Webster). Target contact force was between 7 g and 15 g for all the lesions created. The endpoint of the PVI ablation was the presence of a bidirectional block confirmed by a PENTARAY mapping catheter.

### Temperature monitoring

Throughout the procedure, esophageal temperature was monitored using the CIRCA S-CATH Esophageal Temperature Probe (CIRCA S-CATH, Circa Scientific, Inc). This probe contained 12 temperature sensors and provided temperature monitoring at a rate of 20 measurements per second. An automated alarm system notified the operator each time the esophageal temperature increased to more than 40°C. Since our ablation lesions were for short duration (7 seconds), we did not stop ablating even if temperature increased to >40°C.

### MRI imaging

Prior to cardiac ablation, 11 patients underwent a cardiac MRI for LA structure and LA shape assessment. In addition, the anatomical location and distance of the LA relative to the esophagus was assessed. An LA fibrosis score and its distribution was also obtained from the MRI images in order to guide the therapeutic lesions during cardiac ablation. For those unable to receive a cardiac MRI, a cardiac and PV computed tomography (CT) scan was conducted instead.

### Esophageal endoscopy

All patients had an EGD the day after ablation to look for and evaluate any EI that may have been caused during cardiac ablation. EI was defined as any lesion in the esophagus that had close contact to the LA wall. These lesions were defined as an erythema, ulcer, esophageal bleeding, perforation, or fistula. EGD was performed under moderate anesthesia by an experienced gastroenterologist/endoscopist. Patients with positive endoscopy findings were started on a high-dose proton pump inhibitor for 8 weeks (pantoprazole 40 mg twice daily). Patients with negative endoscopy were discharged on a low-dose proton pump inhibitor (pantoprazole 40 mg daily).

### Statistical analysis

Unless otherwise specified, continuous measurements are summarized as mean ± standard deviation, with categorical variables denoted as frequency counts and percentages of the respective endoscopy result group. Missing values were omitted. Measurements and frequency counts were divided according to endoscopy result. Continuous measurements were tested for equality of central tendency. Both groups’ measurements were tested for normality via the Shapiro-Wilk test. If both did not significantly depart from normality, they were tested for equality of means via an independent 2-sample *t* test; otherwise, a 2-sample Wilcoxon rank sum test was used. Proportionality of categorical variables was tested using Fisher exact test, as some expected cell counts were less than 5.

## Results

### Baseline demographics

A total of 43 patients were included in the study. Mean age was 64.9 years; 46.5% of the patients were female. A total of 62.7% had paroxysmal AF, while the remainder had persistent AF. Of the total study population, 32.5% had a history of coronary artery disease and 11.6% had a history of stroke.

### EGD findings

**Figure 1:**
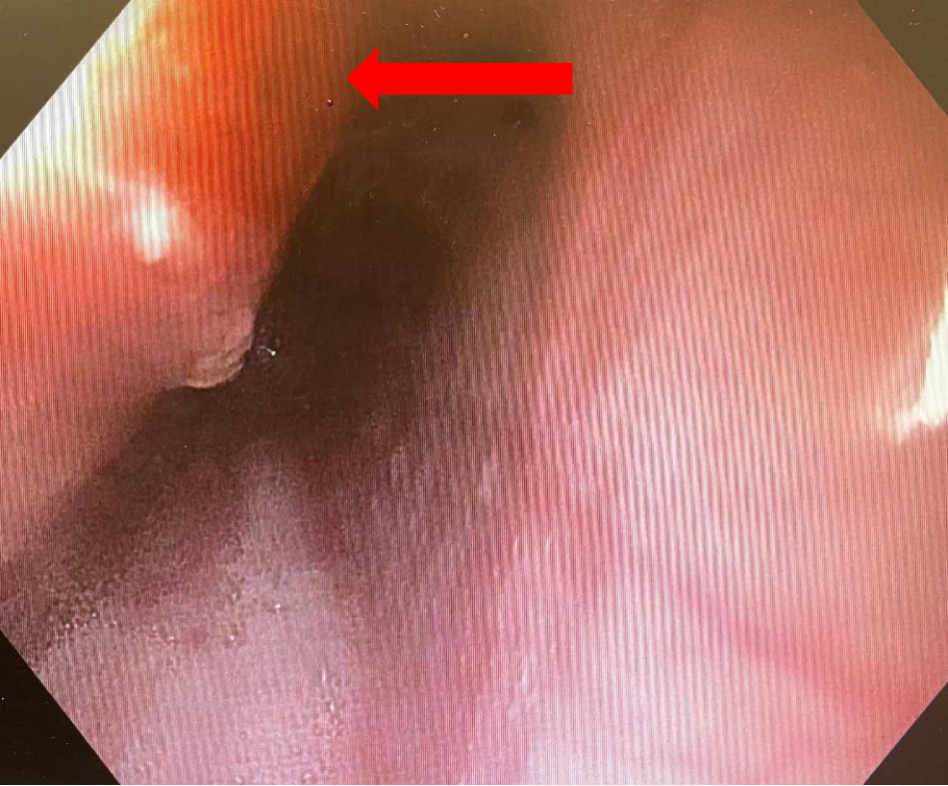
Esophageal ulcer (*arrow*) seen on esophagogastroduodenoscopy.

**Figure 2:**
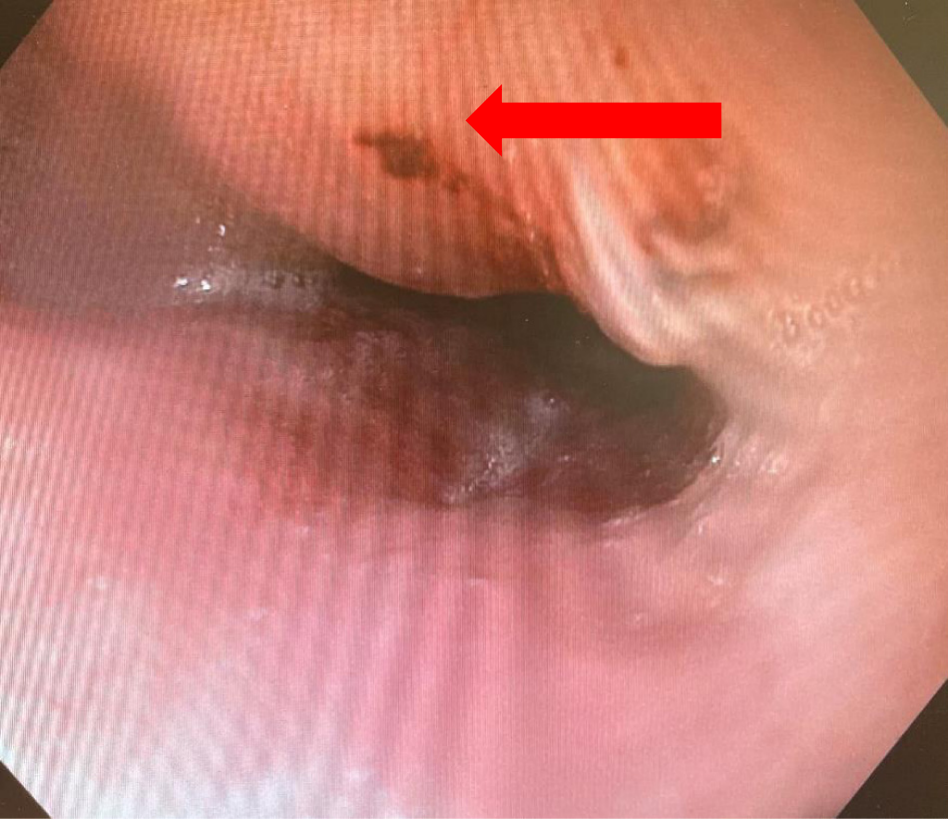
Esophageal erythema (*arrow*) seen on esophagogastroduodenoscopy.

Out of 43 patients, 32 (74%) patients had normal EGD findings (group 1) postablation, whereas 11 (26%) patients had positive findings on EGD following ablation (group 2). EGD findings are shown in Figure 1 and Figure 2. Five lesions were identified as ulcers/erosions of the esophageal mucosa (Figure 1) and five other lesions were of an erythematous nature (Figure 2). All ulcers were noted to be smaller than 5 mm in diameter and all erythematous lesions were localized. One patient showed esophageal mucosal changes suspicious for Barrett esophagus and presented with a bleeding vessel in the mid-esophagus.

Sociodemographic characteristics were similar in both groups: age (66.6 ± 7.4 and 60.1 ± 11.1 years old in group 1 and group 2, respectively, *P* = .095), sex (*P* = .29), and body mass index 35.8 ± 10.3 and 33.6 ± 7.7, respectively (*P* = .47). There was no difference in terms of AF type (persistent or paroxysmal) in both groups (*P* = .49). Comorbidities, such as hypertension (*P* = 1), diabetes (*P* = .71), previous stroke (*P* = .40), heart failure (*P* = .09), and coronary artery disease (*P* = .46), were statistically similar in both groups. All clinical characteristics are summarized in Table 1.

**Table 1:** Clinical characteristics of patients with negative and positive esophageal endoscopy findings

	EGD-negative	EGD-positive	*P* value
N	32	11	
Age, y	66.6 (7.4)	60.1 (11.1)	.0950
BMI	35.8 (10.3)	33.6 (7.7)	.4722
Sex			
Female	13	7	.2947
Male	19	4	
Hypertension	26 (81.2%)	9 (81.8%)	1
Diabetes	9 (28.1%)	4 (36.4%)	.7090
Stroke	3 (9.4%)	2 (18.2%)	.3952
HF	8 (25.0%)	0 (0.0%)	.0900
CAD	9 (28.1%)	5 (15.6%)	.4568
AF type			
Paroxysmal	19	8	.4942
Persistent	13	3	

AF = atrial fibrillation; BMI = body mass index; CAD = coronary artery disease; EGD = esophagogastroduodenoscopy; HF = heart failure; N = number of patients.

### Correlation between temperature and EGD findings

**Figure 3:**
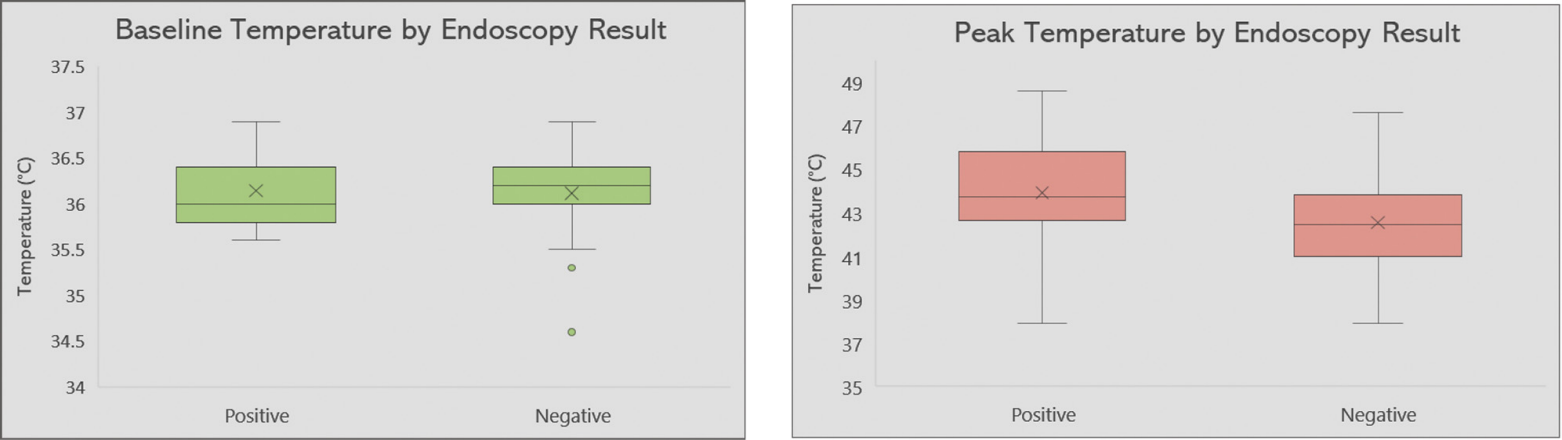
Box plot summarizing the distribution of baseline temperature (**left**) and peak temperature (**right**) in both esophagogastroduodenoscopy-negative and esophagogastroduodenoscopy-positive patients.

The mean esophageal temperature and the highest temperature recorded during ablation were analyzed for each patient. The distribution of both mean temperature and peak temperature are visualized as box plots for both group 1 and group 2 patients (Figure 3). There was no significant difference in mean baseline temperature between both groups (36.1°C ± 0.5°C and 36.1°C ± 0.4°C in EGD-negative and EGD-positive, respectively, *P* = .95). The peak temperature was also similar in both EGD-negative and EGD-positive patients: 42.5°C ± 2.3°C and 43.9°C ± 2.9°C, respectively (*P* = .17). Therefore, we can deduce that there is no significant correlation between the temperatures recorded in the esophagus during ablation and EI post catheter ablation.

### Ablation parameters

**Figure 4:**
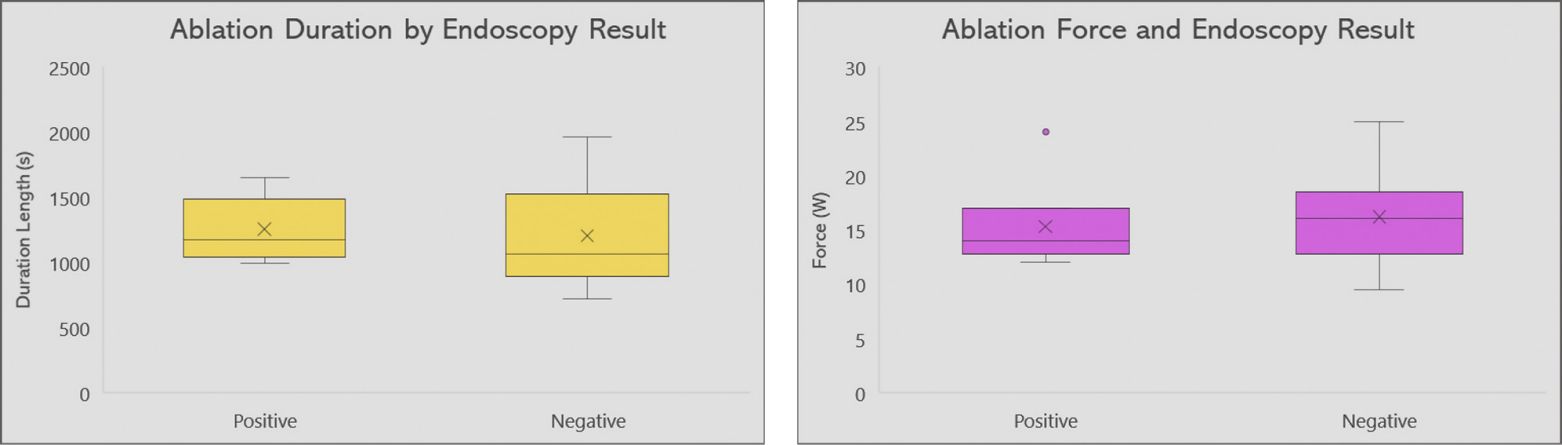
Box plots summarizing the distribution of ablation duration (**left**) and power (**right**) in both esophagogastroduodenoscopy-negative and esophagogastroduodenoscopy-positive patients. EGD = esophagogastroduodenoscopy.

All lesions were created using a high-power (50 W) short-duration (7 seconds) technique. There was no significant difference in both groups regarding total ablation time (1205 ± 373 seconds and 1254 ± 240 seconds in group 1 and group 2 patients, respectively, *P* = .67). In addition, no significant difference was noted regarding the contact force used in both groups (16.2 ± 3.9 g in group 1 and 15.3 ± 3.7 g in group 2 patients, *P* = .53). The correlation between ablation time (A) and contact force (B) in the 2 cohorts is shown in Figure 4.

### MRI and CT scan findings

Out of the 43 subjects, 11 patients received a cardiac MRI prior to catheter ablation. The remaining 25 patients underwent cardiac and PV CT scans. Data summarizing the LA volume, left posterior wall volume, and fibrosis for both groups are shown in Table 2. No significant difference was found between patients without EI and patients who had positive EGD findings in terms of their fibrosis score (20.9%, 95% confidence interval [CI] 14.7–24.3, and 23%, 95% CI 21.8–25.5, respectively; *P* = .56), left atrial volume (114.1 cm^3^, 95% CI 79.3, *P* = .181.2; and 76.8 cm^3^, 95% CI 62.2–91.4, *P* = .29) and posterior wall volume (4.3 cm^3^, 95% CI 3.3–5.8, and 2.8 cm^3^, 95% CI 2.5–3.2, *P* = .19).

**Table 2:** Magnetic resonance imaging / computed tomography findings of patients with negative and positive esophageal endoscopy findings

MRI/CT findings	EGD-negative	EGD-positive	*P* value
Esophageal-LA distance in mm (n = 36 patients)	4.10 (2.9, 5.4)	2.47 (1.69, 3.25)	.0001
Fibrosis score in % (n = 11 patients)	20.9 (14.7, 24.3)	23.7 (21.8, 25.5)	.5557
LA volume in cm^3^ (n = 11 patients)	114.1 (79.3, 181.2)	76.8 (62.2, 91.4)	.2888
LA posterior wall volume in cm^3^ (n = 11 patients)	4.3 (3.3, 5.8)	2.8 (2.5, 3.2)	.1949

CT = computed tomography; EGD = esophagogastroduodenoscopy; LA = left atrial; MRI = magnetic resonance imaging. Values represent mean (95% CI).

**Figure 5:**
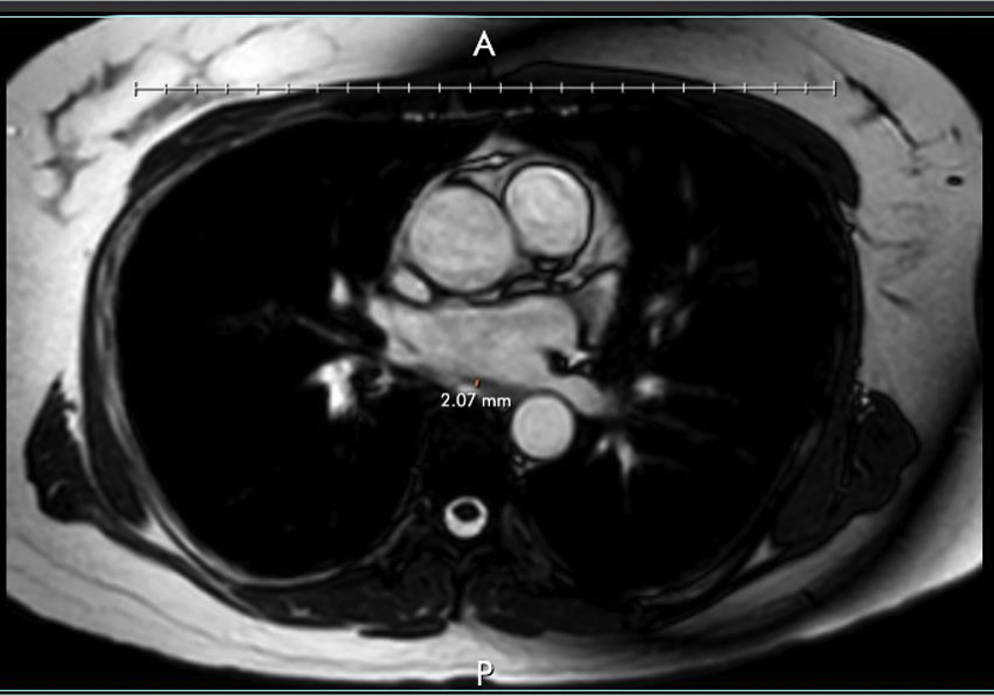
Example of left atrial–esophageal distance measurement based on cardiac magnetic resonance imaging.

Cardiac MRI and CT scans were used to approximate the distance between the LA posterior wall and the esophageal wall. Differences in distance are outlined in Table 2. We observed a significantly smaller esophageal-atrial distance in patients with esophageal anomalies on EGD compared to healthy subjects (*P* = .0001). In fact, an average distance of 2.47 mm was found in the EGD-positive group (95% CI 1.69–3.25) compared to 4.1 mm in the EGD-negative group (95% CI 2.9–5.4). This significantly smaller distance separating the wall of the LA and the esophagus could possibly be associated with a higher incidence of EI after catheter ablation. An example of distance measurement on cardiac MRI is shown in Figure 5.

## Discussion

In this study of 43 patients who underwent HPSD ablation for AF, we demonstrated that the incidence of developing an EI is independent of the highest esophageal temperature reached during ablation. Also, despite a smaller population sample, we observed a strong correlation between the LA-esophagus distance and the occurrence of EI.

### Temperature and ETI

The percentage postablation ETI in our study (26%) was similar to previous findings.^12^ Studies have shown discrepancies on whether temperature is correlated with EI or not. On one hand, Sommer and colleagues^13^ showed that EI depended on the esophageal position and temperature, but not on body mass index. On the other hand, a randomized controlled trial demonstrated that intraesophageal temperature monitoring does not affect the probability of developing EI.

### Relationship of power and duration with ETI

HPSD is a new trend in catheter ablation. Some HPSD benefits include a decrease in fluoroscopy time compared to conventional methods.^14^^,^^15^ It also results in less collateral damage to extracardiac structures owing to reduced resistive heating.^5^ Feasibility and safety of the HSPD procedure has been previously demonstrated in several studies.^16^ A study that included over 10,000 patients (Winkle and colleagues^2^) showed that HPSD ablations had lower complication rates and shorter procedural times compared to other conventional methods. Leshem and colleagues^17^ echoed this finding by demonstrating that HSPD actually improved lesion-to-lesion uniformity, linear contiguity, and transmurality compared to conventional ablation while maintaining a similar safety profile. With specific regard to esophageal injuries, a study conducted by Baher and colleagues^18^ exhibited similar EI rates and patterns in HPSD compared to LPLD ablations when assessed during same-day late gadolinium enhancement MRI. Kaneshiro and colleagues^12^ also showed that while HPSD ablations resulted in higher rates of gastric hypomobility, the prevalence of esophageal lesions as assessed by endoscopy were similar compared to conventional ablation parameters.

A suggestive limitation of HSPD is that it can result in significantly higher temperatures in both the LA and esophageal wall.^19^ However, the correlation as to whether a higher esophageal temperature leads to EI is not well established, thus supplying the necessity of our research study.

In our study, we set the ablation time to 7 seconds per lesion in all our patients, with no exception. A lesion point appears on our CARTO map whenever 7 seconds of ablation are reached. We did not stop ablating even if the temperature increased to >40°C. The reason behind this is to demonstrate that this approach and this specific ablation time are safe and feasible and do not really correlate with EI secondary to high esophageal temperature. Future prospective studies should be conducted comparing 2 arms: the first one with patients to whom we stopped ablating when the temperature reaches >40°C, and the second arm with patients to whom we did not stop ablating.

### ETI in RF ablation: Characteristics and prevention strategies

EI during RF ablation is not a rare occurrence. A recent study showed that the incidence of EI, which included erosions and ulcers after ablation, was 1.3% for each type of lesion.^20^ EI can range from simple esophageal erosions to the more sinister AEF. EI findings may include gastral erosions (22%), esophageal erythema (21%), gastroparesis (17%), hiatal hernia (16%), reflux esophagitis (12%), thermal esophageal lesion (11%), and suspected Barrett esophagus (5%).

Symptoms of EI include chest pain with symptoms of gastroesophageal reflux, hematemesis, fever, hypotension, septic shock, and neurologic symptoms. Currently, there are no clinical guidelines establishing how to prevent EI during catheter ablation procedures. Proton pump inhibitors are routinely given postablation to reduce the risk of an AEF developing by decreasing gastric acidity and gastroesophageal reflux.

Of the many techniques designed to prevent EI, luminal esophageal temperature (LET) monitoring is most commonly used. While earlier studies showed that LET could help potentially reduce EI rates,^21^ more recent investigations have highlighted its limitations. Several independent studies (Ha and colleagues,^22^ Halbfass and colleagues,^23^ Nakagawa and colleagues,^24^ and Kadado and colleagues^8^) all found that LET monitoring was not associated with a reduction in EI rates. Furthermore, 2 other studies analyzed whether EI rates differed when either a multi-thermocouple or a single-sensor probe was used.^25^^,^^26^ They found that although the multi-thermocouple probe had a more sensitive temperature detection, there was ultimately no EI rate difference between the use of the 2 probe types. Barbhaiya and colleagues^22^ emphasized that LET’s major limitation is the necessity for the temperature probe to be within 20 mm of the ablation location. If not kept at that distance, temperature measurements would be considered highly unreliable. Also, other studies have also shown that there is a significant variation in transient thermal response among the various commercially available esophageal temperature probes. Thus, different strategies may lead to an underestimation of luminal esophageal temperature.^27^

Multiple other strategies have been designed to protect the esophagus during catheter ablation, and very few have shown reliable efficacy. A meta-analysis published by Leung and colleagues^28^ demonstrated that esophageal cooling during AF ablation may play a role in reducing the severity of the lesions produced. The IMPACT trial^29^ showed that thermal protection of the esophageal lumen reduces ablation-related thermal injury compared to standard care. However, the IMPACT trial did not provide any evidence that esophageal cooling could in fact protect against AEF development. This shows that the mechanism underlying EI and AEF postablation is not only limited to thermal injury. Bhardwaj and colleagues^30^ used an esophageal balloon retractor to mechanically deviate the esophagus during ablation. This technique is feasible; however, it carries a high risk of mechanical trauma to the esophagus. Multiple studies suggested several anatomical characteristics that could harm the esophagus during ablation and ways to prevent this damage. Sandhu and colleagues^31^ showed that esophageal confinement may be a risk factor for AEF. In addition, Lu and colleagues^32^ proposed that modified posterior-inferior line could serve as a favorable alternative in linear ablation for LA posterior wall isolation.

The pathophysiology underlying EI after ablation remains unclear. Several etiologies include gastric acid reflux, infection, and ischemic injury.^33^ Damage to the esophageal artery during ablation (and therefore ischemic injury) may constitute as one of the primary mechanisms of EI, more so than the likelihood of thermal injury being the cause. This could also explain why AEF presents as a delayed complication postablation.

Although several studies have indicated thermal injury as one of the most important predictors for the development of EI including AEF formation, our investigation indicates no significant correlation between thermal injury and EI. In fact, we found that the most important factor in the development of EI after RF ablation is based on the proximity and anatomic distance between the LA and esophagus. Thus, in case a shorter LA-esophagus distance was found, less energy (<50 W) should be delivered when ablating to an area near the esophagus. Also, the use of an esophageal retractor during the procedure is a feasible and promising technique that may be helpful in avoiding EI.

Bahnson^34^ found that heat transfer during RF ablation depended on the interaction of a multitude of factors, including but not limited to the thickness of the atrial wall, connective tissue, and esophagus, specifically at the “contact-patch.” These interactions are further complicated by intraoperative esophageal and LA movements, making an objective and accurate clinical evaluation more difficult. Despite these complexities, Martinek and colleagues^35^ found several risk factors for esophageal ulcerations. This included patients that had persistent AF; those that received additional lines of ablation at the roofline, LA isthmus, and coronary sinus; and those with LA enlargement. Specifically, patients with LA enlargement are believed to be at higher risk owing to their existing closer contact between the posterior LA wall and the anterior esophageal wall. The mean thickness of these structures measured 2.2 ± 0.9 mm and 3.6 ± 1.7 mm, respectively.^36^ A discontinuous layer of fat lay between the 2 structures, but its thickness was not found to affect ETI rates.^37^ Furthermore, Aupperle and colleagues^37^ found that unipolar RF ablations often produced more intensive and deeper esophageal lesions compared to cryoablation or bipolar RF ablation.

Clinically, our study implicates that when esophageal monitoring was applied, no correlation was seen between EI during PVI ablation and esophageal temperature changes. Larger prospective studies are needed to determine the exact correlation between thermal injury and esophageal lesions, as well as the underlying pathophysiology of lesions’ formation.

### Limitations

Our study has a few limitations. Firstly, the sample study population was small, although it had a power of 0.89. Secondly, our study’s retrospective nature may have limited some of the overall power and precision of this observational study. Therefore, conducting this study prospectively and on a larger scale would provide a more precise correlation between esophageal temperature and esophageal lesions postablation. A randomized controlled trial with 2 arms—1 with esophageal temperature monitoring and 1 without temperature monitoring—should be conducted. The incidence of EI in both arms should be tested to determine if esophageal monitoring during ablation is necessary.

Second, not all the patients were able to receive a cardiac MRI or a CT scan before the procedure. This limited our preassessment of the anatomical relations between the LA and the esophagus, as well as determining the differences in LA volume and posterior wall surface.

## Conclusion

The mechanisms underlying esophageal lesion formation remain to be the result of a multitude of factors. In our study, we determined that during high-power (50 W) short-duration RF ablation, high esophageal temperatures did not directly correlate to a patient’s developing any type of EI.

What we did discover, however, is having a shorter distance between the LA and the esophagus directly correlates to an increased probability of developing EI during cardiac ablation.
